# Supplementary material for: A Current Update on the Distribution, Morphological Features, and Genetic Identity of the Southeast Asian Mahseers, Tor Species
Source: Biology (Basel). 2021 Apr 1;10(4):286. doi: 10.3390/biology10040286 (PMC8065745; doi:10.3390/biology10040286)
Supplement: Supplementary file 1 [file biology-10-00286-s001.pdf]

Supplementary 1

List of selected complete *COX1* gene sequences of *Tor* and *Neolissochilus* species samples obtained from NCBI

No	Accession No.	Species Name	Sequence Length and Size or Position	Origin of Sample	Authors	Ref.
1.	AP011372.1	*T. tambroides*	Complete (bases 6408 to 7958)	Unknown	Miya, M.	[1]
2.	KJ880044.1	*T. tambra*	Complete (bases 6408 to 7958)	Malaysia	Mohamed Yunus, N., Mohd Nor, S.A., Mat Isa, M.N., Lay Kek, T. and Salleh, M.Z.	[2]
3.	KP795444.1	*T. tor*	Complete (bases 5479 to 7029)	India	Sahoo, P.K., Goel, C., Kumar, R. and Barat, A.	[3]
4.	KC914620.1	*T. putitora*	Complete (bases 5480 to 7030)	India	Patiyal, R.S., Sati, J., Barat, A., Sahoo, P.K., Singh, V.K. and Goel, C.	[4]
5.	KJ880045.1	*T. douronensis*	Complete (bases 6410 to 7960)	Malaysia	Mohamed Yunus, N., Mohd Nor, S.A., Mat Isa, M.N., Lay Kek, T. and Salleh, M.Z.	[5]
6.	KF305826.1	*T. sinensis*	Complete (bases 5482 to 7032)	China	Huang, F.J.	[6]
7.	JX444718.1	*T. tambroides*	Complete (bases 6677 to 7954)	Malaysia	Norfatimah, M.Y.	[7]

8.	MN378521.1	*N. hexastichus*	Complete (bases 5479 to 7029)	Unknown	Shubra, S., Pavan-Kumar, A., Archana, M. and Nagpure, N.S.	[8]
9.	MN598560.1	*N. benasi*	Complete (bases 5481 to 7031)	China	Gu, W., Xu, G., Huang, T. and Wang, B.	[9]
10.	NC_031555.1	*N. stracheyi*	Complete (bases 5481 to 7031)	Unknown	Miya, M.	[10]
11.	AP011314.1	*N. soroides*	Complete (bases 5479 to 7029)	Unknown	Miya, M.	[10]
12.	KU380329.1	*N. hexagonolepis*	Complete (bases 5479 to 7029)	China	Zhou, C. and Yuan, D.	[11]

References

1. Nucleotide_[Internet]. *Bethesda (MD): National Library of Medicine (US), National Center for Biotechnology Information; [1988] – . Accession No. AP011372.1, Tor tambroides mitochondrial DNA, complete genome, except for D-loop, DNA*. [cited 2021 30 January]; Available from: https://www.ncbi.nlm.nih.gov/nuccore/AP011372.1.
2. Nucleotide_[Internet]. *Bethesda (MD): National Library of Medicine (US), National Center for Biotechnology Information; [1988] – . Accession No. KJ880044.1, Tor tambra mitochondrion, complete genome, DNA*. Available from: https://www.ncbi.nlm.nih.gov/nuccore/KJ880044.1.
3. Kumar, R., et al., *Complete mitochondrial genome organization of Tor tor (Hamilton, 1822).* Mitochondrial DNA Part A, 2016. **27**(4): p. 2541-2542.
4. Sati, J., et al., *Complete mitochondrial genome organization of Tor putitora*. 2014, Taylor & Francis.
5. Nucleotide_[Internet]. *Bethesda (MD): National Library of Medicine (US), National Center for Biotechnology Information; [1988] – . Accession No. KJ880045.1, Tor douronensis mitochondrion, complete genome, DNA*. Available from: https://www.ncbi.nlm.nih.gov/nuccore/KJ880045.1.
6. Huang, F., et al., *The complete mitochondrial genome sequence of Tor sinensis (Cypriniformes, Cyprinidae).* Mitochondrial DNA, 2015. **26**(5): p. 712-713.
7. Norfatimah, M., et al., *Complete mitochondrial genome of Malaysian Mahseer (Tor tambroides).* Gene, 2014. **548**(2): p. 263-269.
8. Nucleotide_[Internet]. *Bethesda (MD): National Library of Medicine (US), National Center for Biotechnology Information; [1988] – . Accession No. MN378521.1, Neolissochilus hexastichus*

voucher CIFEFGB-SERB-NH mitochondrion, complete genome, DNA. Available from: https://www.ncbi.nlm.nih.gov/nuccore/MN378521.1.
9. Gu, W., et al., *The complete mitochondrial genome of Neolissochilus benasi (Cypriniformes: Cyprinidae).* Mitochondrial DNA Part B, 2020. **5**(1): p. 463-464.
10. Miya, M., *Whole mitochondrial genome sequences in Cypriniformes*. Unpublished manuscript, Natural History Museum & Institute, Chiba, Japan, 2009.
11. Zhou, C., et al., *The complete mitochondrion genome of the Barbodes hexagonolepis (Cypriniformes, cyprinidae).* Mitochondrial DNA Part B, 2016. **1**(1): p. 158-159.

Supplementary 2

List of selected short/partial *COX1* gene sequences of *Tor* species samples obtained from NCBI

No	Accession No.	Species Name and Sample ID	Sequence Length and Size	Origin of Sample	Authors	Ref.
1.	HM536900.1	*T. sinensis* IHBCY0405392	Short/Partial (bases 1 to 685)	China	Yang, L. and Mayden, R.L.	[1]
2.	JF810674.1	*T. douronensis* Hap01	Short/Partial (bases 1 to 452)	Malaysia	Biun, H. and Sade, A.	[2]
3.	JF810675.1	*T. douronensis* Hap02	Short/Partial (bases 1 to 452)	Malaysia	Biun, H. and Sade, A.	[3]
4.	JF810676.1	*T. douronensis* Hap03	Short/Partial (bases 1 to 452)	Malaysia	Biun, H. and Sade, A.	[4]
5.	JF810677.1	*T. douronensis* Hap04	Short/Partial (bases 1 to 452)	Malaysia	Biun, H. and Sade, A.	[5]
6.	JF810678.1	*T. douronensis* Hap05	Short/Partial (bases 1 to 452)	Malaysia	Biun, H. and Sade, A.	[6]
7.	JF810679.1	*T. douronensis* Hap06	Short/Partial (bases 1 to 452)	Malaysia	Biun, H. and Sade, A.	[7]
8.	JF810680.1	*T. douronensis* Hap07	Short/Partial (bases 1 to 452)	Malaysia	Biun, H. and Sade, A.	[8]
9.	JF810681.1	*T. douronensis* Hap08	Short/Partial (bases 1 to 452)	Malaysia	Biun, H. and Sade, A.	[9]
10.	JF957591.1	*T. douronensis* TD1	Short/Partial (bases 1 to 461)	Malaysia	Hassan, N.H., Daud, S.K. and Siraj, S.S.	[10]
11.	JF957592.1	*T. douronensis* TD2	Short/Partial (bases 1 to 461)	Malaysia	Hassan, N.H., Daud, S.K. and Siraj, S.S.	[10]

12.	JF957593.1	*T. douronensis* TD3	Short/Partial (bases 1 to 461)	Malaysia	Hassan, N.H., Daud, S.K. and Siraj, S.S.	[10]
13.	JF957594.1	*T. douronensis* TD4	Short/Partial (bases 1 to 461)	Malaysia	Hassan, N.H., Daud, S.K. and Siraj, S.S.	[10]
14.	JF957595.1	*T. douronensis* TD5	Short/Partial (bases 1 to 461)	Malaysia	Hassan, N.H., Daud, S.K. and Siraj, S.S.	[10]
15.	JF957596.1	*T. douronensis* TD6	Short/Partial (bases 1 to 461)	Malaysia	Hassan, N.H., Daud, S.K. and Siraj, S.S.	[10]
16.	JF957597.1	*T. douronensis* TD7	Short/Partial (bases 1 to 461)	Malaysia	Hassan, N.H., Daud, S.K. and Siraj, S.S.	[10]
17.	JF957598.1	*T. douronensis* TD8	Short/Partial (bases 1 to 461)	Malaysia	Hassan, N.H., Daud, S.K. and Siraj, S.S.	[10]
18.	JF957599.1	*T. douronensis* TD9	Short/Partial (bases 1 to 461)	Malaysia	Hassan, N.H., Daud, S.K. and Siraj, S.S.	[10]
19.	JF957600.1	*T. douronensis* TD10	Short/Partial (bases 1 to 461)	Malaysia	Hassan, N.H., Daud, S.K. and Siraj, S.S.	[10]
20.	JN646100.1	*T. douronensis*	Short/Partial (bases 1 to 672)	Malaysia	Sade, A. and Biun, H.	[11]
21.	KC905001.1	*T. tambroides* 63 F Tarusan	Short/Partial (bases 1 to 703)	Indonesia	Wibowo, A.	[12]
22.	KC905002.1	*T. tambroides* 64 F Tarusan	Short/Partial (bases 1 to 703)	Indonesia	Wibowo, A.	[12]
23.	KC905003.1	*T. tambroides* 65 F Tarusan	Short/Partial (bases 1 to 703)	Indonesia	Wibowo, A.	[12]
24.	KC905004.1	*T. tambroides* 66 F Tarusan	Short/Partial (bases 1 to 703)	Indonesia	Wibowo, A.	[12]
25.	KC905005.1	*T. tambroides* 68 F Tarusan	Short/Partial (bases 1 to 703)	Indonesia	Wibowo, A.	[12]

26.	KC905006.1	*T. tambroides* 69 F Tarusan	Short/Partial (bases 1 to 703)	Indonesia	Wibowo, A.	[12]
27.	KC905007.1	*T. tambroides* 71 F Tarusan	Short/Partial (bases 1 to 703)	Indonesia	Wibowo, A.	[12]
28.	KC905008.1	*T. tambroides* 73 F Lundang	Short/Partial (bases 1 to 703)	Indonesia	Wibowo, A.	[12]
29.	KC905009.1	*T. tambroides* 80 F Lundang	Short/Partial (bases 1 to 703)	Indonesia	Wibowo, A.	[12]
30.	KC905010.1	*T. tambroides* 81 F Lundang	Short/Partial (bases 1 to 703)	Indonesia	Wibowo, A.	[12]
31.	KC905011.1	*T. tambroides* 82 F Lundang	Short/Partial (bases 1 to 703)	Indonesia	Wibowo, A.	[12]
32.	KC905012.1	*T. tambroides* 83 F Lundang	Short/Partial (bases 1 to 703)	Indonesia	Wibowo, A.	[12]
33.	KC905013.1	*T. tambroides* 84 F Lundang	Short/Partial (bases 1 to 703)	Indonesia	Wibowo, A.	[12]
34.	KC905014.1	*T. tambroides* 85 F Lundang	Short/Partial (bases 1 to 703)	Indonesia	Wibowo, A.	[12]
35.	KC905015.1	*T. tambroides* 86 F Lundang	Short/Partial (bases 1 to 703)	Indonesia	Wibowo, A.	[12]
36.	KC905016.1	*T. tambroides* 90 F Ps Minggu	Short/Partial (bases 1 to 703)	Indonesia	Wibowo, A.	[12]
37.	KF240784.1	*T. douronensis* DOFS/MT/7	Short/Partial (bases 1 to 655)	Malaysia	Biun, H., Ibrahim, M.K. and Sade, A.	[13]
38.	KF240785.1	*T. tambroides* DOFS/MT/8	Short/Partial (bases 1 to 655)	Malaysia	Biun, H., Ibrahim, M.K. and Sade, A.	[14]
39.	KJ994634.1	*T. tambra* KIZCXY20090052	Short/Partial (bases 1 to 856)	China	Zheng, L.-P., Yang, J.-X. and Chen, X.-Y.	[15]

40.	KJ994657.1	*T. sinensis* KIZZLP20120103	Short/Partial (bases 1 to 859)	China	Zheng, L.-P., Yang, J.-X. and Chen, X.-Y.	[15]
41.	KT001033.1	*T. tambroides* KLHh29	Short/Partial (bases 1 to 561)	Malaysia	Meganathan, P., Austin, C.M., Tam, S.M., Chew, P.-C., Siow, R., Abdul Rashid, Z. and Song, B.-K.	[16]
42.	KT159237.1	*T. tambra* PHG1 LL	Short/Partial (bases 1 to 609)	Malaysia	Gan, H.M. and Walton, S.	[17]
43.	KT159238.1	*T. tambra* PHG3 LL	Short/Partial (bases 1 to 611)	Malaysia	Gan, H.M. and Walton, S.	[17]
44.	KT159239.1	*T. tambra* PHG4 LL	Short/Partial (bases 1 to 611)	Malaysia	Gan, H.M. and Walton, S.	[17]
45.	KT159240.1	*T. tambra* PHG5 LL	Short/Partial (bases 1 to 611)	Malaysia	Gan, H.M. and Walton, S.	[17]
46.	KT159241.1	*T. tambra* PHG6 LL	Short/Partial (bases 1 to 611)	Malaysia	Gan, H.M. and Walton, S.	[17]
47.	KT159242.1	*T. tambra* PHG7 LL	Short/Partial (bases 1 to 611)	Malaysia	Gan, H.M. and Walton, S.	[17]
48.	KT159243.1	*T. tambra* PHG8 LL	Short/Partial (bases 1 to 611)	Malaysia	Gan, H.M. and Walton, S.	[17]
49.	KT159244.1	*T. tambra* TRG3 LL	Short/Partial (bases 1 to 657)	Malaysia	Gan, H.M. and Walton, S.	[17]
50.	KT159245.1	*T. tambra* TRG4 SL	Short/Partial (bases 1 to 657)	Malaysia	Gan, H.M. and Walton, S.	[17]
51.	KT261292.1	*T. mekongensis* UNS00877	Short/Partial (bases 1 to 584)	Vietnam	Hoang, H.D., Pham, H.M., Durand, J.-D., Trong, N.T. and Phan, P.D.	[18]

52.	KT261293.1	*T. sinensis* UNS00945	Short/Partial (bases 1 to 584)	Vietnam	Hoang, H.D., Pham, H.M., Durand, J.-D., Trong, N.T. and Phan, P.D.	[18]
53.	KT261294.1	*T. sinensis* ZRC 54629	Short/Partial (bases 1 to 584)	Vietnam	Hoang, H.D., Pham, H.M., Durand, J.-D., Trong, N.T. and Phan, P.D.	[18]
54.	KT261295.1	*T. sinensis* UNS00876	Short/Partial (bases 1 to 584)	Vietnam	Hoang, H.D., Pham, H.M., Durand, J.-D., Trong, N.T. and Phan, P.D.	[18]
55.	KT261296.1	*T. sinensis* UNS00614	Short/Partial (bases 1 to 584)	Vietnam	Hoang, H.D., Pham, H.M., Durand, J.-D., Trong, N.T. and Phan, P.D.	[18]
56.	KT261297.1	*T. sinensis* UNS00873	Short/Partial (bases 1 to 584)	Vietnam	Hoang, H.D., Pham, H.M., Durand, J.-D., Trong, N.T. and Phan, P.D.	[18]
57.	KT261298.1	*T. dongnaiensis* UNS00859	Short/Partial (bases 1 to 584)	Vietnam	Hoang, H.D., Pham, H.M., Durand, J.-D., Trong, N.T. and Phan, P.D.	[18]
58.	KT261299.1	*T. dongnaiensis* UNS00889	Short/Partial (bases 1 to 584)	Vietnam	Hoang, H.D., Pham, H.M., Durand, J.-D., Trong, N.T. and Phan, P.D.	[18]
59.	KT261300.1	*T. dongnaiensis* UNS00888	Short/Partial (bases 1 to 584)	Vietnam	Hoang, H.D., Pham, H.M., Durand, J.-D., Trong, N.T. and Phan, P.D.	[18]

60.	KT261301.1	*T. dongnaiensis* ZRC 54627	Short/Partial (bases 1 to 584)	Vietnam	Hoang, H.D., Pham, H.M., Durand, J.-D., Trong, N.T. and Phan, P.D.	[18]
61.	KT354849.1	*T. tambra* Java S2	Short/Partial (bases 1 to 633)	Indonesia	Asih, S. and Walton, S.E.	[17]
62.	KT354850.1	*T. tambra* Java S3	Short/Partial (bases 1 to 633)	Indonesia	Asih, S. and Walton, S.E.	[17]
63.	KT354851.1	*T. tambra* Java S4	Short/Partial (bases 1 to 633)	Indonesia	Asih, S. and Walton, S.E.	[17]
64.	KT354852.1	*T. tambra* Java S5	Short/Partial (bases 1 to 633)	Indonesia	Asih, S. and Walton, S.E.	[17]
65.	KT354853.1	*T. tambra* Java S6	Short/Partial (bases 1 to 633)	Indonesia	Asih, S. and Walton, S.E.	[17]
66.	KT354854.1	*T. tambra* Java S7	Short/Partial (bases 1 to 633)	Indonesia	Asih, S. and Walton, S.E.	[17]
67.	KT354855.1	*T. tambra* Java S8	Short/Partial (bases 1 to 633)	Indonesia	Asih, S. and Walton, S.E.	[17]
68.	KT354856.1	*T. tambra* Java S9	Short/Partial (bases 1 to 633)	Indonesia	Asih, S. and Walton, S.E.	[17]
69.	KT354857.1	*T. tambra* Java S10	Short/Partial (bases 1 to 633)	Indonesia	Asih, S. and Walton, S.E.	[17]
70.	KU692918.1	*T. tambroides* BIF0883	Short/Partial (bases 1 to 536)	Indonesia	Dahruddin, H., Hutama, A., Busson, F., Sauri, S., Hanner, R., Keith, P., Hadiaty, R. and Hubert, N.	[19]

71.	KU692919.1	*T. tambroides* BIF0882	Short/Partial (bases 1 to 512)	Indonesia	Dahruddin, H., Hutama, A., Busson, F., Sauri, S., Hanner, R., Keith, P., Hadiaty, R. and Hubert, N.	[19]

References

1. Yang, L., et al., *Molecular phylogeny of the fishes traditionally referred to Cyprinini sensu stricto (Teleostei: Cypriniformes)*. Zoologica Scripta, 2010. **39**(6): p. 527-550.
2. Nucleotide_[Internet]. *Bethesda (MD): National Library of Medicine (US), National Center for Biotechnology Information; [1988] – . Accession No. JF810674.1, Tor douronensis haplotype Hap01 cytochrome oxidase subunit I (COI) gene, partial cds, mitochondrial, DNA*. Available from: https://www.ncbi.nlm.nih.gov/nuccore/JF810674.1.
3. Nucleotide_[Internet]. *Bethesda (MD): National Library of Medicine (US), National Center for Biotechnology Information; [1988] – . Accession No. JF810675.1, Tor douronensis haplotype Hap02 cytochrome oxidase subunit I (COI) gene, partial cds, mitochondrial, DNA*. Available from: https://www.ncbi.nlm.nih.gov/nuccore/JF810675.1.
4. Nucleotide_[Internet]. *Bethesda (MD): National Library of Medicine (US), National Center for Biotechnology Information; [1988] – . Accession No. JF810676.1, Tor douronensis haplotype Hap03 cytochrome oxidase subunit I (COI) gene, partial cds, mitochondrial, DNA*. Available from: https://www.ncbi.nlm.nih.gov/nuccore/JF810676.1.
5. Nucleotide_[Internet]. *Bethesda (MD): National Library of Medicine (US), National Center for Biotechnology Information; [1988] – . Accession No.JF810677.1, Tor douronensis haplotype Hap04 cytochrome oxidase subunit I (COI) gene, partial cds, mitochondrial, DNA*. Available from: https://www.ncbi.nlm.nih.gov/nuccore/JF810677.1.
6. Nucleotide_[Internet]. *ethesda (MD): National Library of Medicine (US), National Center for Biotechnology Information; [1988] – . Accession No. JF810678.1, Tor douronensis haplotype Hap05 cytochrome oxidase subunit I (COI) gene, partial cds, mitochondrial, DNA*. Available from: https://www.ncbi.nlm.nih.gov/nuccore/JF810678.1.
7. Nucleotide_[Internet]. *Bethesda (MD): National Library of Medicine (US), National Center for Biotechnology Information; [1988] – . Accession No. JF810679.1, Tor douronensis haplotype Hap06 cytochrome oxidase subunit I (COI) gene, partial cds, mitochondrial, DNA*. Available from: https://www.ncbi.nlm.nih.gov/nuccore/JF810679.1.
8. Nucleotide_[Internet]. *Bethesda (MD): National Library of Medicine (US), National Center for Biotechnology Information; [1988] – . Accession No. JF810680.1, Tor douronensis haplotype Hap07 cytochrome oxidase subunit I (COI) gene, partial cds, mitochondrial, DNA*.
9. Nucleotide_[Internet]. *Bethesda (MD): National Library of Medicine (US), National Center for Biotechnology Information; [1988] – . Accession No. JF810681.1, Tor douronensis haplotype*

Hap08 cytochrome oxidase subunit I (COI) gene, partial cds, mitochondrial, DNA. Available from: https://www.ncbi.nlm.nih.gov/nuccore/JF810681.1.
10. Nadiatul, H., et al., *Genetic diversity of Malaysian indigenous Mahseer, Tor douronensis in Sarawak river basins as revealed by cytochrome c oxidase I gene sequences.* Iranian Journal of Animal Biosystematics, 2011. **7**(2).
11. Sadi, A. and H. Biun, *The ichtyofauna of Maliau Basin buzzer zone at Maliau Basin Conservation Area, Sabah, Malaysia.* Journal of Tropical Biology & Conservation (JTBC), 2012.
12. Wibowo, A. and S. Kaban, *GENETIC VARIATION OF Tor tambroides (Bleeker, 1854) ALONG BATANG TARUSAN RIVER, WEST SUMATERA: IMPLICATIONS FOR STOCK IDENTIFICATION.* Indonesian Fisheries Research Journal, 2015. **21**(2): p. 61-66.
13. Nucleotide_[Internet]. *Bethesda (MD): National Library of Medicine (US), National Center for Biotechnology Information; [1988] – . Accession No. KF240784.1, Tor douronensis voucher DOFS/MT/7 cytochrome oxidase subunit I (COI) gene, partial cds, mitochondrial, DNA*. Available from: https://www.ncbi.nlm.nih.gov/nuccore/KF240784.1.
14. Nucleotide_[Internet]. *Bethesda (MD): National Library of Medicine (US), National Center for Biotechnology Information; [1988] – . Accession No. KF240785.1, Tor tambroides voucher DOFS/MT/8 cytochrome oxidase subunit I (COI) gene, partial cds, mitochondrial, DNA*. Available from: https://www.ncbi.nlm.nih.gov/nuccore/KF240785.1.
15. Zheng, L.-P., J.-X. Yang, and X.-Y. Chen, *Molecular phylogeny and systematics of the Barbinae (Teleostei: Cyprinidae) in China inferred from mitochondrial DNA sequences.* Biochemical Systematics and Ecology, 2016. **68**: p. 250-259.
16. Nucleotide_[Internet]. *Bethesda (MD): National Library of Medicine (US), National Center for Biotechnology Information; [1988] – . Accession No. KT001033.1, Tor tambroides isolate KLHh29 cytochrome oxidase subunit I (COI) gene, partial cds, mitochondrial, DNA*. Available from: https://www.ncbi.nlm.nih.gov/nuccore/KT001033.1.
17. Walton, S., et al., *Disentangling the taxonomy of the mahseers (Tor spp.) of Malaysia: An integrated approach using morphology, genetics and historical records.* Reviews in Fisheries Science & Aquaculture, 2017. **25**(3): p. 171-183.
18. Hoang, H.D., et al., *Mahseers genera Tor and Neolissochilus (Teleostei: cyprinidae) from southern Vietnam.* Zootaxa, 2015. **4006**(3): p. 551-568.
19. Dahruddin, H., et al., *Revisiting the ichthyodiversity of Java and Bali through DNA barcodes: taxonomic coverage, identification accuracy, cryptic diversity and identification of exotic species.* Molecular Ecology Resources, 2017. **17**(2): p. 288-299.

Supplementary 3

List of selected complete and short/partial *Cyt b* gene sequences of *Tor* and *Neolissochilus* species samples obtained from NCBI

No	Accession No.	Species Name and Sample ID	Sequence Length and Size/Location	Origin of Sample	Authors	Ref.
1.	KJ880044.1	*T. tambra*	Complete (bases 15301 to 16441)	Malaysia	Mohamed Yunus, N., Mohd Nor, S.A., Mat Isa, M.N., Lay Kek, T. and Salleh, M.Z.	[1]
2.	AP011372.1	*T. tambroides*	Complete (bases 14377 to 15517)	Unknown	Miya, M.	[2]
3.	KP795444.1	*T. tor*	Complete (bases 14367 to 15507)	India	Sahoo, P.K., Goel, C., Kumar, R. and Barat, A.	[3]
4.	KC914620.1	*T. putitora*	Complete (bases 14372 to 15512)	India	Patiyal, R.S., Sati, J., Barat, A., Sahoo, P.K., Singh, V.K. and Goel, C.	[4]
5.	KF305826.1	*T. sinensis*	Complete (bases 14376 to 15516)	China	Huang, F.J.	[5]
6.	KJ880045.1	*T. douronensis*	Complete (bases 15305 to 16445)	Malaysia	Mohamed Yunus, N., Mohd Nor, S.A., Mat Isa, M.N., Lay Kek, T. and Salleh, M.Z.	[6]
7.	JX444718.1	*T. tambroides*	Complete (bases 15297 to 16307	Malaysia	Norfatimah, M.Y., Teh,L.K., Salleh, M.Z.,	[7]

					Mat Isa, M.N. and Siti Azizah ,M.N.	
8.	HM536824.1	*T. tambroides* CBMZF11384	Short/Partial (bases 1 to 1141)	Unknown	Yang, L., Mayden, R.L., Sado, T., He, S., Saitoh, K. and Miya, M.	[8]
9.	EF588160.1	*T. tambroides* TTA04	Short/Partial (bases 1 to 330)	Malaysia	Nguyen, T.T., Na-Nakorn, U., Sukmanomon, S. and Ziming, C.	[9]
10.	EF588161.1	*T. tambroides* TTA03	Short/Partial (bases 1 to 330)	Malaysia	Nguyen, T.T., Na-Nakorn, U., Sukmanomon, S. and Ziming, C.	[9]
11.	EF588163.1	*T. tambroides* TTA07	Short/Partial (bases 1 to 330)	Malaysia	Nguyen, T.T., Na-Nakorn, U., Sukmanomon, S. and Ziming, C.	[9]
12.	EF588166.1	*T. tambroides* TTA05	Short/Partial (bases 1 to 330)	Thailand	Nguyen, T.T., Na-Nakorn, U., Sukmanomon, S. and Ziming, C.	[9]
13.	EF588167.1	*T. tambroides* TTA01	Short/Partial (bases 1 to 330)	Malaysia	Nguyen, T.T., Na-Nakorn, U., Sukmanomon, S. and Ziming, C.	[9]
14.	EF588169.1	*T. tambroides* TTA08	Short/Partial (bases 1 to 330)	Malaysia	Nguyen, T.T., Na-Nakorn, U., Sukmanomon,	[9]

					S. and Ziming, C.	
15.	EF588170.1	*T. tambroides* TTA06	Short/Partial (bases 1 to 330)	Malaysia	Nguyen, T.T., Na-Nakorn, U., Sukmanomon, S. and Ziming, C.	[9]
16.	EF588175.1	*T. tambroides* TTA09	Short/Partial (bases 1 to 330)	Malaysia	Nguyen, T.T., Na-Nakorn, U., Sukmanomon, S. and Ziming, C.	[9]
17.	EF588176.1	*T. tambroides* TTA11	Short/Partial (bases 1 to 330)	Malaysia	Nguyen, T.T., Na-Nakorn, U., Sukmanomon, S. and Ziming, C.	[9]
18.	EF588177. 1	*T. tambroides* TTA12	Short/Partial (bases 1 to 330)	Malaysia	Nguyen, T.T., Na-Nakorn, U., Sukmanomon, S. and Ziming, C.	[9]
19.	EF588178.1	*T. tambroides* TTA14	Short/Partial (bases 1 to 330)	Thailand	Nguyen, T.T., Na-Nakorn, U., Sukmanomon, S. and Ziming, C.	[9]
20.	EF588179.1	*T. tambroides* TTA15	Short/Partial (bases 1 to 330)	Thailand	Nguyen, T.T., Na-Nakorn, U., Sukmanomon, S. and Ziming, C.	[9]
21.	EF588188.1	*T. tambroides* TTA13	Short/Partial (bases 1 to 330)	Indonesia	Nguyen, T.T., Na-Nakorn, U., Sukmanomon,	[9]

					S. and Ziming, C.	
22.	EF588199.1	*T. tambroides* TTA02	Short/Partial (bases 1 to 330)	Thailand	Nguyen, T.T., Na-Nakorn, U., Sukmanomon, S. and Ziming, C.	[9]
23.	EF588197.1	*T. tambroides* TTA10	Short/Partial (bases 1 to 330)	China	Nguyen, T.T., Na-Nakorn, U., Sukmanomon, S. and Ziming, C.	[9]
24.	DQ464985.1	*T. tambroides* CPN 0032	Short/Partial (bases 1 to 573)	Vietnam	Thai, B.T., Ngo, V.S. and Austin, C.M.	[10]
25.	KJ994686.1	*T. tambra* KIZCXY20090052	Short/Partial (bases 1 to 1131)	China	Zheng, L.-P., Yang, J.-X. and Chen, X.-Y.	[11]
26.	DQ366170.1	*T. tambra* TTBSE1	Short/Partial (bases 1 to 408)	Malaysia	Ryan, J.R. and Esa, Y.B.	[12]
27.	KP712155.1	*T. douronensis* CTOL3881	Short/Partial (bases 1 to 1141)	China	Yang, L., Sado, T., Vincent Hirt, M., Pasco-Viel, E., Arunachalam, M., Li, J., Wang, X., Freyhof, J., Saitoh, K., Simons, A.M., Miya, M., He, S. and Mayden, R.L.	[13]
28.	KC696531.1	*T. douronensis*	Short/Partial (bases 1 to 1140)	China	Luo, J., Wang, J., Wu, X.Y., Chen, Z.M., Yue, Z.P., Ma, W. and Chen, S.Y.	[14]

29.	FJ211162.1	*T. douronensis*	Short/Partial (bases 1 to 1122)	China	Guo, B., Tong, C. and He, S.	[15]
30.	EF588149.1	*T. douronensis* TDO01	Short/Partial (bases 1 to 330)	Indonesia	Nguyen, T.T., Na-Nakorn, U., Sukmanomon, S. and Ziming, C.	[9]
31.	EF588150.1	*T. douronensis* TDO06	Short/Partial (bases 1 to 330)	Malaysia	Nguyen, T.T., Na-Nakorn, U., Sukmanomon, S. and Ziming, C.	[9]
32.	EF588151.1	*T. douronensis* TDO05	Short/Partial (bases 1 to 330)	Malaysia	Nguyen, T.T., Na-Nakorn, U., Sukmanomon, S. and Ziming, C.	[9]
33.	EF588152.1	*T. douronensis* TDO10	Short/Partial (bases 1 to 330)	Malaysia	Nguyen, T.T., Na-Nakorn, U., Sukmanomon, S. and Ziming, C.	[9]
34.	EF588153.1	*T. douronensis* TDO11	Short/Partial (bases 1 to 330)	Malaysia	Nguyen, T.T., Na-Nakorn, U., Sukmanomon, S. and Ziming, C.	[9]
35.	EF588154.	1 *T. douronensis* TDO12	Short/Partial (bases 1 to 330)	Malaysia	Nguyen, T.T., Na-Nakorn, U., Sukmanomon, S. and Ziming, C.	[9]
36.	EF588155.1	*T. douronensis* TDO13	Short/Partial (bases 1 to 330)	Malaysia	Nguyen, T.T., Na-Nakorn, U., Sukmanomon,	[9]

					S. and Ziming, C.	
37.	EF588159.1	*T. douronensis* TDO18	Short/Partial (bases 1 to 330)	China	Nguyen, T.T., Na-Nakorn, U., Sukmanomon, S. and Ziming, C.	[9]
38.	EF588168.1	*T. douronensis* TDO09	Short/Partial (bases 1 to 330)	Malaysia	Nguyen, T.T., Na-Nakorn, U., Sukmanomon, S. and Ziming, C.	[9]
39.	EF588171.1	*T. douronensis* TDO08	Short/Partial (bases 1 to 330)	Malaysia	Nguyen, T.T., Na-Nakorn, U., Sukmanomon, S. and Ziming, C.	[9]
40.	EF588185.1	*T. douronensis* TDO07	Short/Partial (bases 1 to 330)	Malaysia	Nguyen, T.T., Na-Nakorn, U., Sukmanomon, S. and Ziming, C.	[9]
41.	EF588186.1	*T. douronensis* TDO03	Short/Partial (bases 1 to 330)	Indonesia	Nguyen, T.T., Na-Nakorn, U., Sukmanomon, S. and Ziming, C.	[9]
42.	EF588187.1	*T. douronensis* TDO04	Short/Partial (bases 1 to 330)	Indonesia	Nguyen, T.T., Na-Nakorn, U., Sukmanomon, S. and Ziming, C.	[9]
43.	EF588189.1	*T. douronensis* TDO02	Short/Partial (bases 1 to 330)	Indonesia	Nguyen, T.T., Na-Nakorn, U., Sukmanomon,	[9]

					S. and Ziming, C.	
44.	EF588190.1	*T. douronensis* TDO14	Short/Partial (bases 1 to 330)	Vietnam	Nguyen, T.T., Na-Nakorn, U., Sukmanomon, S. and Ziming, C.	[9]
45.	EF588191.1	*T. douronensis* TDO15	Short/Partial (bases 1 to 330)	Vietnam	Nguyen, T.T., Na-Nakorn, U., Sukmanomon, S. and Ziming, C.	[9]
46.	EF588194.1	*T. douronensis* TDO16	Short/Partial (bases 1 to 330)	China	Nguyen, T.T., Na-Nakorn, U., Sukmanomon, S. and Ziming, C.	[9]
47.	MN105957.1	*T. tor* H 01	Short/Partial (bases 1 to 1121)	India	Sah, P., Mandal, S., Singh, R.K., Kumar, R., Pathak, A., Dutta, N., Srivastava, J.K., Saini, V.P., Lal, K.K. and Mohindra, V.	[16]
48.	MN105958.1	*T. tor* H 02	Short/Partial (bases 1 to 1121)	India	Sah, P., Mandal, S., Singh, R.K., Kumar, R., Pathak, A., Dutta, N., Srivastava, J.K., Saini, V.P., Lal, K.K. and Mohindra, V.	[16]

49.	MN105959.1	*T. tor* H 03	Short/Partial (bases 1 to 1121)	India	Sah, P., Mandal, S., Singh, R.K., Kumar, R., Pathak, A., Dutta, N., Srivastava, J.K., Saini, V.P., Lal, K.K. and Mohindra, V.	[16]
50.	MN105960.1	*T. tor* H 04	Short/Partial (bases 1 to 1121)	India	Sah, P., Mandal, S., Singh, R.K., Kumar, R., Pathak, A., Dutta, N., Srivastava, J.K., Saini, V.P., Lal, K.K. and Mohindra, V.	[16]
51.	MN105961.1	*T. tor* H 05	Short/Partial (bases 1 to 1121)	India	Sah, P., Mandal, S., Singh, R.K., Kumar, R., Pathak, A., Dutta, N., Srivastava, J.K., Saini, V.P., Lal, K.K. and Mohindra, V.	[16]
52.	MN105962.1	*T. tor* H 06	Short/Partial (bases 1 to 1121)	India	Sah, P., Mandal, S., Singh, R.K., Kumar, R., Pathak, A., Dutta, N., Srivastava, J.K., Saini, V.P., Lal, K.K.	[16]

					and Mohindra, V.	
53.	MN105963.1	*T. tor* H 07	Short/Partial (bases 1 to 1121)	India	Sah, P., Mandal, S., Singh, R.K., Kumar, R., Pathak, A., Dutta, N., Srivastava, J.K., Saini, V.P., Lal, K.K. and Mohindra, V.	[16]
54.	MN105964.1	*T. tor* H 08	Short/Partial (bases 1 to 1121)	India	Sah, P., Mandal, S., Singh, R.K., Kumar, R., Pathak, A., Dutta, N., Srivastava, J.K., Saini, V.P., Lal, K.K. and Mohindra, V.	[16]
55.	MN105965.1	*T. tor* H 09	Short/Partial (bases 1 to 1121)	India	Sah, P., Mandal, S., Singh, R.K., Kumar, R., Pathak, A., Dutta, N., Srivastava, J.K., Saini, V.P., Lal, K.K. and Mohindra, V.	[16]
56.	MN105966.1	*T. tor* H 10	Short/Partial (bases 1 to 1121)	India	Sah, P., Mandal, S., Singh, R.K., Kumar, R., Pathak, A., Dutta, N., Srivastava, J.K., Saini,	[16]

					V.P., Lal, K.K. and Mohindra, V.	
57.	KP712238.1	*T. tor* RLM2879	Short/Partial (bases 1 to 1141)	India	Yang, L., Sado, T., Vincent Hirt, M., Pasco-Viel, E., Arunachalam, M., Li, J., Wang, X., Freyhof, J., Saitoh, K., Simons, A.M., Miya, M., He, S. and Mayden, R.L.	[13]
58.	KP712239.1	*T. tor* RLM2943	Short/Partial (bases 1 to 1141)	India	Yang, L., Sado, T., Vincent Hirt, M., Pasco-Viel, E., Arunachalam, M., Li, J., Wang, X., Freyhof, J., Saitoh, K., Simons, A.M., Miya, M., He, S. and Mayden, R.L.	[13]
59.	KF574626.1	*T. tor* NBFGRTT473	Short/Partial (bases 1 to 1141)	India	Dhawan, S., Lal, K.K., Singh, R.K., Mohindra, V., Chandra, S., Gupta, B.K., Dwivedi, A.K. and Jena, J.K.	[17]
60.	KF574627.1	*T. tor* NBFGRTT474	Short/Partial (bases 1 to 1141)	India	Dhawan, S., Lal, K.K., Singh, R.K., Mohindra, V.,	[17]

					Chandra, S., Gupta, B.K., Dwivedi, A.K. and Jena, J.K.	
61.	KF574628.1	*T. tor* NBFGRTT475	Short/Partial (bases 1 to 1141)	India	Dhawan, S., Lal, K.K., Singh, R.K., Mohindra, V., Chandra, S., Gupta, B.K., Dwivedi, A.K. and Jena, J.K.	[17]
62.	KF574629.1	*T. tor* NBFGRTT476	Short/Partial (bases 1 to 1141)	India	Dhawan, S., Lal, K.K., Singh, R.K., Mohindra, V., Chandra, S., Gupta, B.K., Dwivedi, A.K. and Jena, J.K.	[17]
63.	KF574630.1	*T. tor* NBFGRTT479	Short/Partial (bases 1 to 1141)	India	Dhawan, S., Lal, K.K., Singh, R.K., Mohindra, V., Chandra, S., Gupta, B.K., Dwivedi, A.K. and Jena, J.K.	[17]
64.	KF574631.1	*T. tor* NBFGRTT480	Short/Partial (bases 1 to 1141)	India	Dhawan, S., Lal, K.K., Singh, R.K., Mohindra, V., Chandra, S., Gupta, B.K., Dwivedi, A.K. and Jena, J.K.	[17]
65.	KF574632.1	*T. tor* NBFGRTT481	Short/Partial (bases 1 to 1141)	India	Dhawan, S., Lal, K.K., Singh, R.K., Mohindra, V., Chandra, S., Gupta, B.K.,	[17]

					Dwivedi, A.K. and Jena, J.K.	
66.	KF574633.1	*T. tor* NBFGRTT482	Short/Partial (bases 1 to 1141)	India	Dhawan, S., Lal, K.K., Singh, R.K., Mohindra, V., Chandra, S., Gupta, B.K., Dwivedi, A.K. and Jena, J.K.	[17]
67.	KC696530.1	*T. sinensis*	Short/Partial (bases 1 to 1140)	China	Luo, J., Wang, J., Wu, X.Y., Chen, Z.M., Yue, Z.P., Ma, W. and Chen, S.Y.	[14]
68.	HM536802.1	*T. sinensis* IHBCY0405392	Short/Partial (bases 1 to 1141)	China	Yang, L., Mayden, R.L., Sado, T., He, S., Saitoh, K. and Miya, M.	[8]
69.	FJ211164.1	*T. sinensis*	Short/Partial (bases 1 to 1122)	China	Guo, B., Tong, C. and He, S.	[15]
70.	KJ994709.1	*T. sinensis* KIZZLP20120103	Short/Partial (bases 1 to 1131)	China	Zheng, L.-P., Yang, J.-X. and Chen, X.-Y.	[11]
71.	KP998091.1	*T. putitora* TP1	Short/Partial (bases 1 to 843)	India	Neelesh, D., Marcus Knight, J.D., Mandar, P.S., Ashwin, R., Ronald, D.K.P., Anvar Ali, P.H., Unmesh, K., Rajeev, R. and Siby, P.	[18]
72.	KP998067.1	*T. putitora* TP2	Short/Partial (bases 1 to 952)	India	Neelesh, D., Marcus Knight, J.D., Mandar, P.S., Ashwin, R.,	[19]

					Ronald, D.K.P., Anvar Ali, P.H., Unmesh, K., Rajeev, R. and Siby, P.	
73.	KX234729.1	*T. putitora* MF19	Short/Partial (bases 1 to 418)	India	Laskar, B.A., Kundu, S., Tyagi, K. and Kumar, V.	[20]
74.	KX234728.1	*T. putitora* MF18	Short/Partial (bases 1 to 418)	India	Laskar, B.A., Kundu, S., Tyagi, K. and Kumar, V.	[21]
75.	KX234727.1	*T. putitora* MF17	Short/Partial (bases 1 to 418)	India	Laskar, B.A., Kundu, S., Tyagi, K. and Kumar, V.	[22]
76.	KU524917.1	*T. putitora* SMF31305	Short/Partial (bases 1 to 1141)	Unknown	Borkenhagen, K.	[23]
77.	KU524918.1	*T. putitora* SMF31306	Short/Partial (bases 1 to 1141)	Unknown	Borkenhagen, K.	[23]
78.	KU524916.1	*T. putitora* SMF31304	Short/Partial (bases 1 to 1141)	Unknown	Borkenhagen, K.	[23]
79.	KP712187.1	*T. putitora* CTOL3933	Short/Partial (bases 1 to 1141)	Nepal	Yang, L., Sado, T., Vincent Hirt, M., Pasco-Viel, E., Arunachalam, M., Li, J., Wang, X., Freyhof, J., Saitoh, K., Simons, A.M., Miya, M., He, S. and Mayden, R.L.	[13]

80.	KF574491.1	*T. putitora* NBFGRTP1708	Short/Partial (bases 1 to 1141)	India	Lal, K.K., Singh, R.K., Mohindra, V., Dhawan, S., Chandra, S., Gupta, B.K., Kumar, R., Sah, R.S. and Jena, J.K.	[17]
81.	KF574492.1	*T. putitora* NBFGRTP1709	Short/Partial (bases 1 to 1141)	India	Lal, K.K., Singh, R.K., Mohindra, V., Dhawan, S., Chandra, S., Gupta, B.K., Kumar, R., Sah, R.S. and Jena, J.K.	[17]
82.	KF574493.1	*T. putitora* NBFGRTP1710	Short/Partial (bases 1 to 1141)	India	Lal, K.K., Singh, R.K., Mohindra, V., Dhawan, S., Chandra, S., Gupta, B.K., Kumar, R., Sah, R.S. and Jena, J.K.	[17]
83.	KF574494.1	*T. putitora* NBFGRTP1711	Short/Partial (bases 1 to 1141)	India	Lal, K.K., Singh, R.K., Mohindra, V., Dhawan, S., Chandra, S., Gupta, B.K., Kumar, R., Sah, R.S. and Jena, J.K.	[17]
84.	KF574495.1	*T. putitora* NBFGRTP1712	Short/Partial (bases 1 to 1141)	India	Lal, K.K., Singh, R.K., Mohindra, V., Dhawan, S., Chandra, S., Gupta, B.K., Kumar, R.,	[17]

					Sah, R.S. and Jena, J.K.	
85.	KF574496.1	*T. putitora* NBFGRTP1714	Short/Partial (bases 1 to 1141)	India	Lal, K.K., Singh, R.K., Mohindra, V., Dhawan, S., Chandra, S., Gupta, B.K., Kumar, R., Sah, R.S. and Jena, J.K.	[17]
86.	KF574497.1	*T. putitora* NBFGRTP1715	Short/Partial (bases 1 to 1141)	India	Lal, K.K., Singh, R.K., Mohindra, V., Dhawan, S., Chandra, S., Gupta, B.K., Kumar, R., Sah, R.S. and Jena, J.K.	[17]
87.	KF574498.1	*T. putitora* NBFGRTP1716	Short/Partial (bases 1 to 1141)	India	Lal, K.K., Singh, R.K., Mohindra, V., Dhawan, S., Chandra, S., Gupta, B.K., Kumar, R., Sah, R.S. and Jena, J.K.	[17]
88.	KF574499.1	*T. putitora* NBFGRTP1717	Short/Partial (bases 1 to 1141)	India	Lal, K.K., Singh, R.K., Mohindra, V., Dhawan, S., Chandra, S., Gupta, B.K., Kumar, R., Sah, R.S. and Jena, J.K.	[17]
89.	KF574500.1	*T. putitora* NBFGRTP1718	Short/Partial (bases 1 to 1141)	India	Lal, K.K., Singh, R.K., Mohindra, V., Dhawan, S., Chandra, S.,	[17]

					Gupta, B.K., Kumar, R., Sah, R.S. and Jena, J.K.	
90.	KF574501.1	*T. putitora* NBFGRTP1720	Short/Partial (bases 1 to 1141)	India	Lal, K.K., Singh, R.K., Mohindra, V., Dhawan, S., Chandra, S., Gupta, B.K., Kumar, R., Sah, R.S. and Jena, J.K.	[17]
91.	MN378521.1	*N. hexastichus*	Complete (bases 14373 to 15513)	Unknown	Shubra, S., Pavan-Kumar, A., Archana, M. and Nagpure, N.S.	[24]
92.	MN598560.1	*N. benasi*	Complete (bases 14375 to 15515)	China	Gu, W., Xu, G., Huang, T. and Wang, B.	[25]
93.	NC_031555.1	*N. stracheyi*	Complete (bases 14374 to 15514)	Unknown	Miya, M.	[26]
94.	AP011314.1	*N. soroides*	Complete (bases 14372 to 15512)	Unknown	Miya, M.	[26]
95.	KU380329.1	*N. hexagonolepis*	Complete (bases 14372 to 15512)	Unknown	Zhou, C. and Yuan, D.	[27]

References

1. Nucleotide_[Internet]. *Bethesda (MD): National Library of Medicine (US), National Center for Biotechnology Information; [1988] – . Accession No. KJ880044.1, Tor tambra mitochondrion, complete genome, DNA*. Available from: https://www.ncbi.nlm.nih.gov/nuccore/KJ880044.1.
2. Nucleotide_[Internet]. *Bethesda (MD): National Library of Medicine (US), National Center for Biotechnology Information; [1988] – . Accession No. AP011372.1, Tor tambroides mitochondrial DNA, complete genome, except for D-loop, DNA*. [cited 2021 30 January]; Available from: https://www.ncbi.nlm.nih.gov/nuccore/AP011372.1.
3. Kumar, R., et al., *Complete mitochondrial genome organization of Tor tor (Hamilton, 1822).* Mitochondrial DNA Part A, 2016. **27**(4): p. 2541-2542.

4. Sati, J., et al., *Complete mitochondrial genome organization of Tor putitora*. 2014, Taylor & Francis.
5. Huang, F., et al., *The complete mitochondrial genome sequence of Tor sinensis (Cypriniformes, Cyprinidae).* Mitochondrial DNA, 2015. **26**(5): p. 712-713.
6. Nucleotide_[Internet]. *Bethesda (MD): National Library of Medicine (US), National Center for Biotechnology Information; [1988] – . Accession No. KJ880045.1, Tor douronensis mitochondrion, complete genome, DNA*. Available from: https://www.ncbi.nlm.nih.gov/nuccore/KJ880045.1.
7. Norfatimah, M., et al., *Complete mitochondrial genome of Malaysian Mahseer (Tor tambroides).* Gene, 2014. **548**(2): p. 263-269.
8. Yang, L., et al., *Molecular phylogeny of the fishes traditionally referred to Cyprinini sensu stricto (Teleostei: Cypriniformes).* Zoologica Scripta, 2010. **39**(6): p. 527-550.
9. Nguyen, T.T., et al., *A study on phylogeny and biogeography of mahseer species (Pisces: Cyprinidae) using sequences of three mitochondrial DNA gene regions.* Molecular Phylogenetics and Evolution, 2008. **3**(48): p. 1223-1231.
10. Nucleotide_[Internet]. *Bethesda (MD): National Library of Medicine (US), National Center for Biotechnology Information; [1988] – . Accession No. DQ464985.1, Tor tambroides voucher CPN 0032 cytochrome b gene, partial cds, mitochondrial, DNA*. Available from: https://www.ncbi.nlm.nih.gov/nuccore/DQ464985.1.
11. Zheng, L.-P., J.-X. Yang, and X.-Y. Chen, *Molecular phylogeny and systematics of the Barbinae (Teleostei: Cyprinidae) in China inferred from mitochondrial DNA sequences.* Biochemical Systematics and Ecology, 2016. **68**: p. 250-259.
12. Esa, Y.B., et al., *Phylogenetic relationships among several freshwater fishes (Family: Cyprinidae) in Malaysia inferred from partial sequencing of the cytochrome b mitochondrial DNA (mtDNA) gene.* Pertanika J Trop Agric Sci, 2012. **35**: p. 307-318.
13. Yang, L., et al., *Phylogeny and polyploidy: resolving the classification of cyprinine fishes (Teleostei: Cypriniformes).* Molecular Phylogenetics and Evolution, 2015. **85**: p. 97-116.
14. Wang, J., et al., *Molecular phylogeny of European and African Barbus and their West Asian relatives in the Cyprininae (Teleostei: Cypriniformes) and orogenesis of the Qinghai-Tibetan Plateau.* Chinese Science Bulletin, 2013. **58**(31): p. 3738-3746.
15. Guo, B., C. Tong, and S. He, *Sox genes evolution in closely related young tetraploid cyprinid fishes and their diploid relative.* Gene, 2009. **439**(1-2): p. 102-112.
16. Sah, P., et al., *Genetic structure of natural populations of endangered Tor mahseer, Tor tor (Hamilton, 1822) inferred from two mitochondrial DNA markers.* Meta Gene, 2020. **23**: p. 100635.
17. Lal, K., et al., *Molecular characterization of Indian freshwater Cyprinids using cytochrome C Oxidase I sequences.* National Bureau of Fish Conservation Division, NBFGR, Uttar Pradesh, India, 2014.
18. Nucleotide_[Internet]. *Bethesda (MD): National Library of Medicine (US), National Center for Biotechnology Information; [1988] – . Accession No. KP998091.1, Tor putitora isolate TP1 cytochrome b (cytb) gene, partial cds, mitochondrial, DNA*. Available from: https://www.ncbi.nlm.nih.gov/nuccore/KP998091.1.

19. Nucleotide_[Internet]. *Bethesda (MD): National Library of Medicine (US), National Center for Biotechnology Information; [1988] – . Accession No. KP998067.1, Tor putitora isolate TP2 cytochrome b (cytb) gene, partial cds, mitochondrial, DNA*. Available from: https://www.ncbi.nlm.nih.gov/nuccore/KP998067.1.
20. Nucleotide_[Internet]. *Bethesda (MD): National Library of Medicine (US), National Center for Biotechnology Information; [1988] – . Accession No. KX234729.1, Tor putitora voucher MF19 cytochrome b (cytb) gene, partial cds, mitochondrial. DNA*. Available from: https://www.ncbi.nlm.nih.gov/nuccore/KX234729.1.
21. Nucleotide_[Internet]. *Bethesda (MD): National Library of Medicine (US), National Center for Biotechnology Information; [1988] – . Accession No. KX234728.1, Tor putitora voucher MF18 cytochrome b (cytb) gene, partial cds, mitochondrial, DNA*. Available from: https://www.ncbi.nlm.nih.gov/nuccore/KX234728.1.
22. Nucleotide_[Internet]. *Bethesda (MD): National Library of Medicine (US), National Center for Biotechnology Information; [1988] – . Accession No. KX234727.1, Tor putitora voucher MF17 cytochrome b (cytb) gene, partial cds, mitochondrial, DNA*. Available from: https://www.ncbi.nlm.nih.gov/nuccore/KX234727.1.
23. Borkenhagen, K., *Molecular phylogeny of the tribe Torini Karaman, 1971 (Actinopterygii: Cypriniformes) from the Middle East and North Africa.* Zootaxa, 2017. **4236**(2): p. zootaxa.4236.2.4.
24. Nucleotide_[Internet]. *Bethesda (MD): National Library of Medicine (US), National Center for Biotechnology Information; [1988] – . Accession No. MN378521.1, Neolissochilus hexastichus voucher CIFEFGB-SERB-NH mitochondrion, complete genome, DNA*. Available from: https://www.ncbi.nlm.nih.gov/nuccore/MN378521.1.
25. Gu, W., et al., *The complete mitochondrial genome of Neolissochilus benasi (Cypriniformes: Cyprinidae).* Mitochondrial DNA Part B, 2020. **5**(1): p. 463-464.
26. Miya, M., *Whole mitochondrial genome sequences in Cypriniformes.* Unpublished manuscript, Natural History Museum & Institute, Chiba, Japan, 2009.
27. Zhou, C., et al., *The complete mitochondrion genome of the Barbodes hexagonolepis (Cypriniformes, cyprinidae).* Mitochondrial DNA Part B, 2016. **1**(1): p. 158-159.

Supplementary 4

List of selected complete and short/partial *16S rRNA* gene sequences of *Tor* and *Neolissochilus* species samples obtained from NCBI

No	Accession No.	Species Name and Sample ID	Sequence Length and Size/Location	Origin of Sample	Authors	Ref.
1.	MG229067.1	*T. putitora* TP-04/16S/AJK/2017	Short/Partial (bases 1 to 1677)	Unknown	Maqsood, I., Shafi, N., Ali, G. and Akhtar, T.	[1]
2.	MG229066.1	*T. putitora* TP-03/16S/AJK/2017	Short/Partial (bases 1 to 1677)	Unknown	Maqsood, I., Shafi, N., Ali, G. and Akhtar, T.	[2]
3.	MG229065.1	*T. putitora* TP-02/16S/AJK/2017	Short/Partial (bases 1 to 1677)	Unknown	Maqsood, I., Shafi, N., Ali, G. and Akhtar, T.	[3]
4.	MG229064.1	*T. putitora* TP-01/16S/AJK/2017	Short/Partial (bases 1 to 1677)	Unknown	Maqsood, I., Shafi, N., Ali, G. and Akhtar, T.	[4]
5.	KX925208.1	*T. sinensis*	Short/Partial (bases 1 to 622)	China	Li, Y.-Z., Fan, L.-X. and Li, X.-S.	[5]
6.	KP795444.1	*T. tor*	Complete (bases 1098 to 2774)	India	Kumar, R., Goel, C., Kumari Sahoo, P., Singh, A.K. and Barat, A.	[6]
7.	KP712696.1	*T. tor* RLM2943	Short/Partial (bases 1 to 591)	India	Yang, L., Sado, T., Vincent Hirt, M., Pasco-Viel, E., Arunachalam, M., Li,J., Wang, X., Freyhof,J.,	[7]

					Saitoh, K., Simons, A.M., Miya, M., He, S. and Mayden, R.L.	
8.	KP712695.1	*T. tor* RLM2879	Short/Partial (bases 1 to 564)	India	Yang, L., Sado, T., Vincent Hirt, M., Pasco-Viel, E., Arunachalam, M., Li,J., Wang, X., Freyhof,J., Saitoh, K., Simons, A.M., Miya, M., He, S. and Mayden, R.L.	[7]
9.	KP712645.1	*T. putitora* CTOL3933	Short/Partial (bases 1 to 562)	Nepal	Yang, L., Sado, T., Vincent Hirt, M., Pasco-Viel, E., Arunachalam, M., Li,J., Wang, X., Freyhof,J., Saitoh, K., Simons, A.M., Miya, M., He, S. and Mayden, R.L.	[7]
10.	KJ880045.1	*T. douronensis* (Complete)	Complete (bases 2022 to 3699)	Malaysia	Mohamed Yunus, N., Mohd Nor, S.A., Mat Isa, M.N., Lay Kek, T. and Salleh, M.Z.	[8]
11.	KJ880044.1	*T. tambra* (Complete)	Complete (bases 2024 to 3697)	Malaysia	Mohamed Yunus, N., Mohd Nor,	[9]

					S.A., Mat Isa, M.N., Lay Kek, T. and Salleh, M.Z.	
12.	KJ869423.1	*T. putitora* TPF	Short/Partial (bases 1 to 572)	India	Agarwal, N.K. and Chaudary, D.K.	[10]
13.	KF305826.1	*T. sinensis* (Complete)	Complete (bases 1098 to 2776)	China	Huang, F.J.	[11]
14.	KC914620.1	*T. putitora* (Complete)	Complete (bases 1099 to 2773)	India	Patiyal, R.S., Sati, J., Barat, A., Sahoo, P.K., Singh, V.K. and Goel, C.	[12]
15.	JX444718.1	*T. tambroides* (Complete)	Complete (bases 2020 to 3693)	Malaysia	Norfatimah, M.Y., Teh, L.K., Salleh, M.Z., Mat Isa, M.N. and Siti Azizah, M.N.	[13]
16.	JX204402.1	*T. putitora* Tp16s10	Short/Partial (bases 1 to 550)	India	Barat, A., Pandey, H. and Singh, B.K.	[14]
17.	JX204401.1	*T. putitora* Tp16s9	Short/Partial (bases 1 to 550)	India	Barat, A., Pandey, H. and Singh, B.K.	[14]
18.	JX204400.1	*T. putitora* Tp16s8	Short/Partial (bases 1 to 550)	India	Barat, A., Pandey, H. and Singh, B.K.	[14]
19.	JX204399.1	*T. putitora* Tp16s7	Short/Partial (bases 1 to 550)	India	Barat, A., Pandey, H. and Singh, B.K.	[14]
20.	JX204398.1	*T. putitora* Tp16s6	Short/Partial (bases 1 to 550)	India	Barat, A., Pandey, H.	[14]

					and Singh, B.K.	
21.	JX204397.1	*T. putitora* Tp16s5	Short/Partial (bases 1 to 550)	India	Barat, A., Pandey, H. and Singh, B.K.	[14]
22.	JX204396.1	*T. putitora* Tp16s4	Short/Partial (bases 1 to 550)	India	Barat, A., Pandey, H. and Singh, B.K.	[14]
23.	JX204395.1	*T. putitora* Tp16s3	Short/Partial (bases 1 to 550)	India	Barat, A., Pandey, H. and Singh, B.K.	[14]
24.	JX204394.1	*T. putitora* Tp16s2	Short/Partial (bases 1 to 550)	India	Barat, A., Pandey, H. and Singh, B.K.	[14]
25.	JX204393.1	*T. putitora* Tp16s1	Short/Partial (bases 1 to 550)	India	Barat, A., Pandey, H. and Singh, B.K.	[14]
26.	JX090196.1	*T. tor* cellline TTH	Short/Partial (bases 1 to 578)	India	Yadav, K., Lakra, W.S., Sharma, J., Goswami, M. and Singh, A.	[15]
27.	JN032124.1	*T. tor* cellline TTCF	Short/Partial (bases 1 to 578)	India	Yadav, K., Lakra, W.S., Sharma, J., Goswami, M. and Singh, A.	[16]
28.	HM536781.1	*T. tambroides* CBMZF11384	Short/Partial (bases 1 to 591)	Unknown	Yang, L., Mayden, R.L., Sado, T., He, S., Saitoh, K. and Miya, M.	[17]
29.	EF588088.1	*T. putitora* TPU01	Short/Partial (bases 1 to 536)	Nepal	Nguyen, T.T., Na-Nakorn, U., Sukmanomon, S. and Ziming, C.	[18]

30.	EF588087.1	*T. tambroides* TTA02	Short/Partial (bases 1 to 536)	Thailand	Nguyen, T.T., Na-Nakorn, U., Sukmanomon, S. and Ziming, C.	[18]
31.	EF588086.1	*T. douronensis* TDO20	Short/Partial (bases 1 to 536)	China	Nguyen, T.T., Na-Nakorn, U., Sukmanomon, S. and Ziming, C.	[18]
32.	EF588085.1	*T. tambroides* TTA10	Short/Partial (bases 1 to 536)	China	Nguyen, T.T., Na-Nakorn, U., Sukmanomon, S. and Ziming, C.	[18]
33.	EF588084.1	*T. douronensis* TDO19	Short/Partial (bases 1 to 536)	China	Nguyen, T.T., Na-Nakorn, U., Sukmanomon, S. and Ziming, C.	[18]
34.	EF588083.1	*T. douronensis* TDO17	Short/Partial (bases 1 to 536)	China	Nguyen, T.T., Na-Nakorn, U., Sukmanomon, S. and Ziming, C.	[18]
35.	EF588082.1	*T. douronensis* TDO16	Short/Partial (bases 1 to 536)	China	Nguyen, T.T., Na-Nakorn, U., Sukmanomon, S. and Ziming, C.	[18]
36.	EF588079.1	*T. douronensis* TDO15	Short/Partial (bases 1 to 535)	Vietnam	Nguyen, T.T., Na-Nakorn, U., Sukmanomon, S. and Ziming, C.	[18]

37.	EF588078.1	*T. douronensis* TDO14	Short/Partial (bases 1 to 536)	Vietnam	Nguyen, T.T., Na-Nakorn, U., Sukmanomon, S. and Ziming, C.	[18]
38.	EF588077.1	*T. douronensis* TDO02	Short/Partial (bases 1 to 536)	Indonesia	Nguyen, T.T., Na-Nakorn, U., Sukmanomon, S. and Ziming, C.	[18]
39.	EF588076.1	*T. tambroides* TTA13	Short/Partial (bases 1 to 536)	Indonesia	Nguyen, T.T., Na-Nakorn, U., Sukmanomon, S. and Ziming, C.	[18]
40.	EF588075.1	*T. douronensis* TDO04	Short/Partial (bases 1 to 537)	Indonesia	Nguyen, T.T., Na-Nakorn, U., Sukmanomon, S. and Ziming, C.	[18]
41.	EF588074.1	*T. douronensis* TDO03	Short/Partial (bases 1 to 537)	Indonesia	Nguyen, T.T., Na-Nakorn, U., Sukmanomon, S. and Ziming, C.	[18]
42.	EF588073.1	*T. douronensis* TDO07	Short/Partial (bases 1 to 537)	Malaysia	Nguyen, T.T., Na-Nakorn, U., Sukmanomon, S. and Ziming, C.	[18]
43.	EF588072.1	*T. tor* TTO02	Short/Partial (bases 1 to 536)	Bangladesh	Nguyen, T.T., Na-Nakorn, U., Sukmanomon, S. and Ziming, C.	[18]

44.	EF588071.1	*T. putitora* TPU06	Short/Partial (bases 1 to 536)	Bangladesh	Nguyen, T.T., Na-Nakorn, U., Sukmanomon, S. and Ziming, C.	[18]
45.	EF588068.1	*T. tor* TTO01	Short/Partial (bases 1 to 536)	Nepal	Nguyen, T.T., Na-Nakorn, U., Sukmanomon, S. and Ziming, C.	[18]
46.	EF588067.1	*T. tambroides* TTA15	Short/Partial (bases 1 to 536)	Thailand	Nguyen, T.T., Na-Nakorn, U., Sukmanomon, S. and Ziming, C.	[18]
47.	EF588066.1	*T. tambroides* TTA14	Short/Partial (bases 1 to 536)	Thailand	Nguyen, T.T., Na-Nakorn, U., Sukmanomon, S. and Ziming, C.	[18]
48.	EF588065.1	*T. tambroides* TTA12	Short/Partial (bases 1 to 536)	Malaysia	Nguyen, T.T., Na-Nakorn, U., Sukmanomon, S. and Ziming, C.	[18]
49.	EF588064.1	*T. tambroides* TTA11	Short/Partial (bases 1 to 536)	Malaysia	Nguyen, T.T., Na-Nakorn, U., Sukmanomon, S. and Ziming, C.	[18]
50.	EF588063.1	*T. tambroides* TTA09	Short/Partial (bases 1 to 536)	Malaysia	Nguyen, T.T., Na-Nakorn, U., Sukmanomon, S. and Ziming, C.	[18]

51.	EF588061.1	*T. putitora* TPU04	Short/Partial (bases 1 to 536)	Nepal	Nguyen, T.T., Na-Nakorn, U., Sukmanomon, S. and Ziming, C.	[18]
52.	EF588060.1	*T. putitora* TPU05	Short/Partial (bases 1 to 536)	Nepal	Nguyen, T.T., Na-Nakorn, U., Sukmanomon, S. and Ziming, C.	[18]
53.	EF588059.1	*T. douronensis* TDO08	Short/Partial (bases 1 to 537)	Malaysia	Nguyen, T.T., Na-Nakorn, U., Sukmanomon, S. and Ziming, C.	[18]
54.	EF588058.1	*T. tambroides* TTA06	Short/Partial (bases 1 to 536)	Malaysia	Nguyen, T.T., Na-Nakorn, U., Sukmanomon, S. and Ziming, C.	[18]
55.	EF588057.1	*T. tambroides* TTA01	Short/Partial (bases 1 to 536)	Thailand	Nguyen, T.T., Na-Nakorn, U., Sukmanomon, S. and Ziming, C.	[18]
56.	EF588056.1	*T. tambroides* TTA05	Short/Partial (bases 1 to 536)	Thailand	Nguyen, T.T., Na-Nakorn, U., Sukmanomon, S. and Ziming, C.	[18]
57.	EF588053.1	*T. tambroides* TTA07	Short/Partial (bases 1 to 536)	Malaysia	Nguyen, T.T., Na-Nakorn, U., Sukmanomon, S. and Ziming, C.	[18]

58.	EF588051.1	*T. tambroides* TTA03	Short/Partial (bases 1 to 536)	Malaysia	Nguyen, T.T., Na-Nakorn, U., Sukmanomon, S. and Ziming, C.	[18]
59.	EF588050.1	*T. tambroides* TTA04	Short/Partial (bases 1 to 536)	Malaysia	Nguyen, T.T., Na-Nakorn, U., Sukmanomon, S. and Ziming, C.	[18]
60.	EF588049.1	*T. douronensis* TDO18	Short/Partial (bases 1 to 536)	China	Nguyen, T.T., Na-Nakorn, U., Sukmanomon, S. and Ziming, C.	[18]
61.	EF588046.1	*T. douronensis* TDO05	Short/Partial (bases 1 to 537)	Malaysia	Nguyen, T.T., Na-Nakorn, U., Sukmanomon, S. and Ziming, C.	[18]
62.	EF588045.1	*T. douronensis* TDO06	Short/Partial (bases 1 to 537)	Malaysia	Nguyen, T.T., Na-Nakorn, U., Sukmanomon, S. and Ziming, C.	[18]
63.	EF588044.1	*T. douronensis* TDO01	Short/Partial (bases 1 to 536)	Indonesia	Nguyen, T.T., Na-Nakorn, U., Sukmanomon, S. and Ziming, C.	[18]
64.	DQ845877.1	*T. douronensis*	Short/Partial (bases 1 to 1665)	China	Li, J., Wang, X., Kong, X., Zhao, K., He, S. and Mayden, R.L.	[19]

65.	DQ845876.1	*T. sinensis*	Short/Partial (bases 1 to 1641)	China	Li, J., Wang, X., Kong, X., Zhao, K., He, S. and Mayden, R.L.	[19]
66.	DQ464925.1	*T. douronensis* CPN 0030	Short/Partial (bases 1 to 494)	Vietnam	Thai, B.T., Austin, C.M. and Ngo, V.S.	[20]
67.	DQ464914.1	*T. tambroides* CPN 0032	Short/Partial (bases 1 to 494)	Vietnam	Thai, B.T., Austin, C.M. and Ngo, V.S.	[21]
68.	AY973165.1	*T. douronensis* DUOSAR 03	Short/Partial (bases 1 to 542)	Malaysia	Nguyen, T.T.T., Ingram, B., Sungan, S., Gooley, G., Sim, S.Y., Tinggi, D.	[22]
69.	AY973164.1	*T. douronensis* DUOSAR 02	Short/Partial (bases 1 to 542)	Malaysia	Nguyen, T.T.T., Ingram, B., Sungan, S., Gooley, G., Sim, S.Y., Tinggi, D.	[22]
70.	AY973163.1	*T. douronensis* DUOSAR 01	Short/Partial (bases 1 to 542)	Malaysia	Nguyen, T.T.T., Ingram, B., Sungan, S., Gooley, G., Sim, S.Y., Tinggi, D.	[22]
71.	AY973160.1	*T. tambroides* TAMSAR 04	Short/Partial (bases 1 to 541)	Malaysia	Nguyen, T.T.T., Ingram, B., Sungan, S., Gooley, G., Sim, S.Y., Tinggi, D.	[22]
72.	AY973159.1	*T. tambroides* TAMSAR 03	Short/Partial (bases 1 to 541)	Malaysia	Nguyen, T.T.T., Ingram, B., Sungan, S.,	[22]

					Gooley, G., Sim, S.Y., Tinggi, D.	
73.	AY973158.1	*T. tambroides* TAMSAR 02	Short/Partial (bases 1 to 541)	Malaysia	Nguyen, T.T.T., Ingram, B., Sungan, S., Gooley, G., Sim, S.Y., Tinggi, D.	[22]
74.	AY973157.1	*T. tambroides* TAMSAR 01	Short/Partial (bases 1 to 540)	Malaysia	Nguyen, T.T.T., Ingram, B., Sungan, S., Gooley, G., Sim, S.Y., Tinggi, D.	[22]
75.	AP011372.1	*T. tambroides*	Complete (bases 1099 to 2770)	Unknown	Miya, M.	[23]
76.	MN378521.1	*N. hexastichus*	Complete (bases 1098 to 2776)	Unknown	Shubra, S., Pavan-Kumar, A., Archana, M. and Nagpure, N.S.	[24]
77.	MN598560.1	*N. benasi*	Complete (bases 1099 to 2771)	China	Gu, W., Xu, G., Huang, T. and Wang, B.	[25]
78.	NC_031555.1	*N. stracheyi*	Complete (bases 1099 to 2771)	Unknown	Miya, M.	[26]
79.	AP011314.1	*N. soroides*	Complete (bases 1098 to 2776)	Unknown	Miya, M.	[26]
80.	KU380329.1	*N. hexagonolepis*	Complete (bases 1098 to 2776)	China	Zhou, C., Yuan, D., Zhu, C., Lei, L., Zhang, C., Zhou, J., Gong, J., Zhu, L., Li, B. and Wu, Q.	[27]

References

1. Nucleotide_[Internet]. *Bethesda (MD): National Library of Medicine (US), National Center for Biotechnology Information; [1988] – . Accession No. MG229067.1, Tor putitora isolate TP-04/16S/AJK/2017 16S ribosomal RNA gene, partial sequence, mitochondrial. DNA*. Available from: https://www.ncbi.nlm.nih.gov/nuccore/MG229067.1.
2. Nucleotide_[Internet]. *Bethesda (MD): National Library of Medicine (US), National Center for Biotechnology Information; [1988] – . Accession No. MG229066.1, Tor putitora isolate TP-03/16S/AJK/2017 16S ribosomal RNA gene, partial sequence, mitochondrial, DNA*. Available from: https://www.ncbi.nlm.nih.gov/nuccore/MG229066.1.
3. Nucleotide_[Internet]. *Bethesda (MD): National Library of Medicine (US), National Center for Biotechnology Information; [1988] – . Accession No. MG229065.1, Tor putitora isolate TP-02/16S/AJK/2017 16S ribosomal RNA gene, partial sequence, mitochondrial, DNA*. Available from: https://www.ncbi.nlm.nih.gov/nuccore/MG229065.1.
4. *Bethesda (MD): National Library of Medicine (US), National Center for Biotechnology Information; [1988] – . Accession No. MG229064.1, Tor putitora isolate TP-01/16S/AJK/2017 16S ribosomal RNA gene, partial sequence, mitochondrial, DNA*. Available from: https://www.ncbi.nlm.nih.gov/nuccore/MG229064.1.
5. Nucleotide_[Internet]. *Bethesda (MD): National Library of Medicine (US), National Center for Biotechnology Information; [1988] – . Accession No. KX925208.1, Tor sinensis 16S ribosomal RNA gene, partial sequence, mitochondrial, DNA*. Available from: https://www.ncbi.nlm.nih.gov/nuccore/KX925208.1.
6. Kumar, R., et al., *Complete mitochondrial genome organization of Tor tor (Hamilton, 1822).* Mitochondrial DNA Part A, 2016. **27**(4): p. 2541-2542.
7. Yang, L., et al., *Phylogeny and polyploidy: resolving the classification of cyprinine fishes (Teleostei: Cypriniformes).* Molecular Phylogenetics and Evolution, 2015. **85**: p. 97-116.
8. Nucleotide_[Internet]. *Bethesda (MD): National Library of Medicine (US), National Center for Biotechnology Information; [1988] – . Accession No. KJ880045.1, Tor douronensis mitochondrion, complete genome, DNA*. Available from: https://www.ncbi.nlm.nih.gov/nuccore/KJ880045.1.
9. Nucleotide_[Internet]. *Bethesda (MD): National Library of Medicine (US), National Center for Biotechnology Information; [1988] – . Accession No. KJ880044.1, Tor tambra mitochondrion, complete genome, DNA*. Available from: https://www.ncbi.nlm.nih.gov/nuccore/KJ880044.1.
10. Nucleotide_[Internet]. *Bethesda (MD): National Library of Medicine (US), National Center for Biotechnology Information; [1988] – . Accession No. KJ869423.1, Tor putitora isolate TPF 16S ribosomal RNA gene, partial sequence, mitochondrial, DNA*. Available from: https://www.ncbi.nlm.nih.gov/nuccore/KJ869423.1.
11. Huang, F., et al., *The complete mitochondrial genome sequence of Tor sinensis (Cypriniformes, Cyprinidae).* Mitochondrial DNA, 2015. **26**(5): p. 712-713.
12. Sati, J., et al., *Complete mitochondrial genome organization of Tor putitora*. 2014, Taylor & Francis.
13. Norfatimah, M., et al., *Complete mitochondrial genome of Malaysian Mahseer (Tor tambroides).* Gene, 2014. **548**(2): p. 263-269.

14. Pandey, H., et al., *Comparative Phylogenetic Study of four Coldwater Fishes (Family Cyprinidae) Based on Targeted 16S RN A Mitochondrial Gene.* Journal of Ecophysiology and Occupational Health, 2013. **13**(3/4): p. 47.
15. Nucleotide_[Internet]. *Bethesda (MD): National Library of Medicine (US), National Center for Biotechnology Information; [1988] – . Accession No. JX090196.1, Tor tor cell-line TTH 16S ribosomal RNA gene, partial sequence, mitochondrial, DNA*. Available from: https://www.ncbi.nlm.nih.gov/nuccore/JX090196.1.
16. Yadav, K., et al., *Development and characterization of a cell line TTCF from endangered mahseer Tor tor (Ham.).* Fish physiology and biochemistry, 2012. **38**(4): p. 1035-1045.
17. Yang, L., et al., *Molecular phylogeny of the fishes traditionally referred to Cyprinini sensu stricto (Teleostei: Cypriniformes).* Zoologica Scripta, 2010. **39**(6): p. 527-550.
18. Nguyen, T.T., et al., *A study on phylogeny and biogeography of mahseer species (Pisces: Cyprinidae) using sequences of three mitochondrial DNA gene regions.* Molecular Phylogenetics and Evolution, 2008. **3**(48): p. 1223-1231.
19. Li, J., et al., *Variation patterns of the mitochondrial 16S rRNA gene with secondary structure constraints and their application to phylogeny of cyprinine fishes (Teleostei: Cypriniformes).* Molecular Phylogenetics and Evolution, 2008. **47**(2): p. 472-487.
20. Nucleotide_[Internet]. *Bethesda (MD): National Library of Medicine (US), National Center for Biotechnology Information; [1988] – . Accession No. DQ464925.1, Tor douronensis voucher CPN 0030 16S ribosomal RNA gene, partial sequence, mitochondrial, DNA*. Available from: https://www.ncbi.nlm.nih.gov/nuccore/DQ464925.1.
21. Nucleotide_[Internet]. *Bethesda (MD): National Library of Medicine (US), National Center for Biotechnology Information; [1988] – . Accession No. DQ464914.1, Tor tambroides voucher CPN 0032 16S ribosomal RNA gene, partial sequence, mitochondrial, DNA*. Available from: https://www.ncbi.nlm.nih.gov/nuccore/DQ464914.1.
22. Nguyen, T.T., et al., *Mitochondrial DNA diversity of broodstock of two indigenous mahseer species, Tor tambroides and T. douronensis (Cyprinidae) cultured in Sarawak, Malaysia.* Aquaculture, 2006. **253**(1-4): p. 259-269.
23. Nucleotide_[Internet]. *Bethesda (MD): National Library of Medicine (US), National Center for Biotechnology Information; [1988] – . Accession No. AP011372.1, Tor tambroides mitochondrial DNA, complete genome, except for D-loop, DNA*. [cited 2021 30 January]; Available from: https://www.ncbi.nlm.nih.gov/nuccore/AP011372.1.
24. Nucleotide_[Internet]. *Bethesda (MD): National Library of Medicine (US), National Center for Biotechnology Information; [1988] – . Accession No. MN378521.1, Neolissochilus hexastichus voucher CIFEFGB-SERB-NH mitochondrion, complete genome, DNA*. Available from: https://www.ncbi.nlm.nih.gov/nuccore/MN378521.1.
25. Gu, W., et al., *The complete mitochondrial genome of Neolissochilus benasi (Cypriniformes: Cyprinidae).* Mitochondrial DNA Part B, 2020. **5**(1): p. 463-464.
26. Miya, M., *Whole mitochondrial genome sequences in Cypriniformes.* Unpublished manuscript, Natural History Museum & Institute, Chiba, Japan, 2009.
27. Zhou, C., et al., *The complete mitochondrion genome of the Barbodes hexagonolepis (Cypriniformes, cyprinidae).* Mitochondrial DNA Part B, 2016. **1**(1): p. 158-159.
